# Collection of implementation-related data in pragmatic clinical trials: a cross-sectional study from the NIH Pragmatic Trials Collaboratory

**DOI:** 10.1186/s43058-026-00868-4

**Published:** 2026-02-13

**Authors:** Katy E. Trinkley, Shruti Gohil, Stacie A. Salsbury, Diana J. Burgess, Ardith Z. Doorenbos, Brian S. Mittman, Wynne E. Norton, Hayden B. Bosworth, Karen L. Staman, Devon Check

**Affiliations:** 1 University of Colorado School of Medicine Department of Family Medicine, Anschutz Medical Campus, Aurora, CO USA; 2 University of California Irvine School of Medicine, Irvine, CA USA; 3 Palmer Center for Chiropractic Research, Palmer College of Chiropractic, Davenport, IA USA; 4 Center for Care Delivery and Outcomes Research, Minneapolis VA Health Care System, Minneapolis, MN USA; 5 University of Minnesota Medical School, Minneapolis, USA; 6 University of Illinois, College of Nursing, Chicago, IL USA; 7 Kaiser Permanente Southern California, Pasadena, CA USA; 8 Division of Cancer Control and Population Sciences, National Cancer Institute, Bethesda, MD USA; 9 Duke University Department of Population Health Sciences, Durham, NC USA; 10 Duke Clinical Research Institute, Durham, NC USA; 11 University of Michigan Medical School, Ann Arbor, MI USA

**Keywords:** Pragmatic clinical trial, Implementation science, Costs, Sustainability

## Abstract

**Background:**

Embedded pragmatic clinical trials (ePCTs) are conducted as part of routine care, which provides researchers and health systems multiple opportunities to study implementation processes and outcomes.

**Methods:**

We conducted a cross-sectional survey of 32 ePCTs associated with the NIH Pragmatic Trials Collaboratory to assess the implementation-related outcomes that were measured or were planned to be measured, including reach (number and percent of eligible patients who participate in an intervention and the representativeness of those patients), patient engagement in or adherence to the intervention, adoption (number and percent of eligible organizations or clinicians that decide to take up or use an intervention), fidelity (clinician’s delivery of an intervention as intended), adaptations (changes or modifications to an intervention), sustainability (potential for an intervention to be maintained or institutionalized after a trial concludes), sustainment (actual maintenance or institutionalization of an intervention after a trial concludes), and costs. The trials represented different phases of progress (planned, ongoing, or completed).

**Results:**

91% of study teams completed the survey, and most (86%) reported measuring reach. The total number of teams measuring other outcomes was 76% for adherence, 45% for clinician adoption, 93% for fidelity, 69% for adaptations, 24% for sustainability, 38% for sustainment, and 31% for costs.

**Conclusion:**

There is an opportunity for growth in measuring clinician adoption of the intervention, sustainability, sustainment, and associated costs. Measurement of these constructs in future ePCTs could result in development of improved implementation strategies to increase the likelihood of effective implementation leading to equitable, sustainable, and scalable improvement in practice.

**Supplementary Information:**

The online version contains supplementary material available at 10.1186/s43058-026-00868-4.

Contributions to the literature

- Pragmatic clinical trials offer unique opportunities to study implementation processes and outcomes because the tested interventions are embedded into usual clinical workflows and routine care practices.
- While measurement of some constructs (reach, adherence, fidelity, and adaptations) was high across the trials, evaluation of costs, clinician adoption and representativeness, and anticipated and actual sustainment of tested interventions was low.
- To benefit from the substantial investment into pragmatic clinical trials, we need to improve measurement of constructs that drive the implementation of evidence into routine care, including information about costs, sustainability, and sustainment.

## Background

Embedded pragmatic clinical trials (ePCTs) are conducted in routine clinical care settings and conditions, leveraging existing health system infrastructure to assess the effectiveness of health interventions in everyday practice. By design, ePCTs provide important insights and generate data on how well interventions work when delivered in usual clinical workflows and practices in order to provide information relevant to decision makers, including patients, clinicians, and policy makers [1]. Because ePCTs are conducted in the context of routine care, they also provide an ideal opportunity to collect data on how the intervention is implemented in addition to the impact of the intervention on improving health outcomes [2]. Understanding implementation barriers, facilitators, and processes can increase the probability of continued use of the intervention in routine-care settings as well as how to expand the delivery of such interventions to other clinical contexts.

Since 2012, the National Institutes of Health (NIH) Pragmatic Trials Collaboratory (‘Collaboratory’) has supported 32 large-scale ePCTs designed to address major public health issues, such as non-opioid treatments for chronic pain [3–7], clinical decision support for antibiotic treatments [8, 9] chronic disease medication adherence [10], and advance care planning [11, 12]. (Collaboratory trial descriptions are available at https://rethinkingclinicaltrials.org/demonstration-projects/.) Collaboratory ePCTs are conducted under the NIH two-phase UG3/UH3 cooperative agreement award mechanism. The UG3 award consists of a one- or two-year planning phase, and the UH3 phase is a four-year phase for conducting the trial. All Collaboratory trials are conducted in partnership with healthcare systems, where tested interventions are embedded into usual clinical workflows and practices. Recognizing the potential for ePCTs to provide important insights on implementation, the Collaboratory has augmented its focus on clinical effectiveness to study implementation as well. For example, earlier Notice of Funding Opportunities (NOFOs) from the Collaboratory (2012 to 2017) called for “efficient, large-scale pragmatic clinical trials [13].” By 2018, the NOFOs sought applications for “efficient, large-scale embedded pragmatic or implementation trials [14].”

Given this context, the Collaboratory has a unique opportunity to draw on wide-ranging project experiences to generate lessons learned that inform best practices for implementation research within ePCTs. To date, several Collaboratory ePCTs have published process evaluations that include information relevant to “real world” implementation of study findings [15–18]. However, the Collaboratory has yet to evaluate how and to what extent ePCTs collect and analyze data to inform the implementation of effective interventions into everyday clinical care settings following study completion. To that end, we conducted a survey to assess the current inclusion of implementation-related research activities in ePCTs to guide improved attention to implementation issues in ongoing and future trials.

## Methods

This cross-sectional study was approved by the Duke University Institutional Review Board (Pro00085360-AMD-9.0, December 2, 2024). Co-leads from the NIH Pragmatic Trials Collaboratory’s Implementation Science Core and working group members developed survey items to assess the extent to which ePCTs evaluated implementation determinants and outcomes. The Implementation Science Core includes representatives from many of the ePCTs surveyed who use a variety of different frameworks in their studies. Item development was guided by their experience with established implementation science frameworks, primarily the RE-AIM framework (Reach, Effectiveness, Adoption, Implementation, and Maintenance) [19], Proctor et al.'s taxonomy of implementation outcomes (e.g., acceptability, feasibility, fidelity, and sustainability), [20] and the Consolidated Framework for Implementation Research (CFIR), which emphasizes multilevel determinants such as inner and outer context, intervention characteristics, and process [21]. Additional guidance came from the Promoting Action in Research Implementation in Health Services (PARiHS) framework [22], and the Practical, Robust Implementation and Sustainability Model (PRISM) [23], which emphasizes sustainability and practical fit in healthcare systems.

**Fig. 1:**
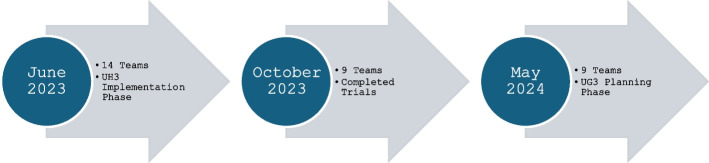
Data collection waves by trial implementation phase

The resulting 28-item survey assessed both implementation outcomes (e.g., fidelity, adherence, sustainability) and determinants (e.g., organizational context, leadership support, and resource availability). The instrument included both structured checklist items and open-ended responses, asking respondents to describe their data collection methods, such as measures used, the timing of assessments, and which partner roles or perspectives were represented. Slight variations in wording were used to reflect project phase; investigators of ongoing trials (UG3/UH3 phase) were asked to report planned measurement activities, while those from completed studies were asked to describe how implementation outcomes and determinants were assessed (see Appendix 1). The survey was piloted by team members and administered anonymously via Qualtrics (Seattle, WA, USA). We invited all 32 ePCTs affiliated with the NIH Pragmatic Trials Collaboratory from 2012 to 2024 to participate. These included 9 completed, 14 ongoing, and 9 recently funded studies. Invitations were sent in 3 waves aligned with trial phase from June 2023 through May 2024 (see Fig. 1). Surveys were sent to principal investigators (PIs), were completed by the PI or designated team member (e.g., co-investigator or project manager) and were summarized descriptively overall and by trial phase.

## Results

Representatives from 29 of 32 trials completed the survey (91% response rate). The 29 trials represented included 7 of 9 completed trials, 13 of 14 ongoing (UH3) trials, and all 9 of the planning phase (UG3) trials. Table 1 reports the implementation constructs evaluated by each trial. The distribution of outcome selection varied across studies (Table 2); some studies only collected one outcome, and others collected all 9 of the outcomes we asked about.

**Table 1:** Implementation constructs measured by trial phase

Implementation Construct Questions	Planning Phase (UG3) Trials *n* = 9	Ongoing (UH3) Trials *n* = 13	Completed Trials *n *= 7	Total N (%) Trials *N* = 29
Are you measuring or planning to measure **reach** (number and percent of eligible patients who participate in an intervention and the representativeness of those patients)?	8 (89%)	10 (77%)	7 (100%)	25 (86%)
Are you measuring or planning to measure patients’ engagement in or **adherence** to the intervention?	7 (78%)	9 (69%)	6 (86%)	23 (79%)
Are you measuring or planning to measure **adoption** (number and percent of eligible organizations or clinicians that decide to take up or use an intervention)?	3 (33%)	5 (38%)	5 (71%)	13 (45%)
Are you measuring or planning to measure **fidelity** (clinician’s delivery of an intervention as intended)?	8 (89%)	12 (92%)	7 (100%)	27 (93%)
Are you measuring or planning to measure **adaptations** (changes or modifications to an intervention)?	5 (60%)	10 (77%)	5 (71%)	20 (69%)
Are you measuring or planning to measure anticipated **sustainability** (potential for an intervention to be maintained or institutionalized after a trial concludes)	3 (33%)	2 (15%)	2 (29%)	7 (24%)
Are you planning to measure known/actual **sustainment** (actual maintenance or institutionalization of an intervention after a trial concludes)?	2 (22%)	4 (31%)	4 (57%)	11 (38%)
Are you collecting or planning to collect data on **costs** associated with the intervention?	4 (40%)	4 (30%)	2 (29%)	9 (31%)
Are you assessing or planning to assess **barriers to and/or facilitators** of implementing (i.e., delivering, using, engaging with) the intervention in the context of the trial?	9 (100%)	12 (92%)	6 (86%)	27 (93%)

**Table 2:** Outcome distribution across studies

Trial	Reach	Adherence	Adoption	Fidelity	Adaptations	Sustainability	Sustainment	Cost	Barriers/ Facilitators	**TOTAL**
**Planning Phase Trials (*n* = 9)**										
1	Y	N	N	Y	I	I	I	Y	Y	4/9 (44%)
2	Y	Y	I	Y	Y	I	I	N	Y	6/9 (67%)
3	Y	N	Y	Y	Y	Y	N	Y	Y	6/9 (67%)
4	I	Y	I	Y	I	I	I	N	Y	3/9 (33%)
5	Y	Y	N	Y	Y	Y	Y	N	Y	7/9 (78%)
6	Y	Y	N	Y	N	I	I	N	Y	4/9 (44%)
7	Y	Y	I	I	I	I	I	Y	Y	4/9 (44%)
8	Y	Y	Y	Y	Y	I	I	N	Y	6/9 (67%)
9	Y	Y	Y	Y	Y	Y	Y	Y	Y	9/9 (100%)
**Total**	8	7	3	8	5	3	2	4	9	
**Ongoing Trials (*n* = 13)**										
1	Y	Y	N	Y	Y	Y	N	Y	Y	7/9 (78%)
2	Y	N	Y	Y	Y	N	N	N	Y	5/9 (56%)
3	Y	Y	N	Y	Y	N	Y	N	Y	6/9 (67%)
4	N	Y	N	Y	Y	N	N	N	Y	4/9 (44%)
5	Y	Y	Y	Y	Y	N	N	N	Y	6/9 (67%)
6	Y	Y	Y	Y	Y	N	Y	Y	Y	7/9 (78%)
7	Y	Y	N	Y	N	N	N	N	Y	4/9 (44%)
8	Y	N	Y	Y	Y	N	N	Y	Y	7/9 (78%)
9	N	N	N	Y	N	N	N	N	Y	2/9 (22%)
10	Y	Y	Y	Y	Y	Y	Y	N	Y	8/9 (89%)
11	Y	Y	N	Y	Y	N	N	N	Y	5/9 (56%)
12	N	N	N	N	N	N	Y	N	N	1/9 (11%)
13	Y	Y	N	Y	Y	N	N	Y	Y	5/9 (56%)
**Total**	10	9	5	12	10	2	4	4	12	
**Completed Trials (*n* = 7)**										
1	Y	Y	Y	Y	Y	Y	Y	Y	Y	9/9 (100%)
2	Y	Y	Y	Y	Y	N	Y	Y	Y	8/9 (89%)
3	Y	Y	Y	Y	Y	N	Y	M	M	3/9 (33%)
4	Y	Y	N	Y	N	N	N	N	Y	4/9 (44%)
5	Y	Y	N	Y	N	N	N	N	Y	4/9 (44%)
6	Y	N	Y	Y	Y	Y	Y	N	Y	7/9 (78%)
P7	Y	Y	Y	Y	Y	N	N	N	Y	6/9 (67%)
**Total**	7 (100%)	6	5	7	5	2	4	2	6	
**All Trials (*n* = 29)**										
**Total**	25 (86%)	22 (76%)	13 (45%)	27 (93%)	20 (69%)	7 (24%)	10 (34%)	10 (34%)	27 (93%)	

*Y* yes, *I *interested to measure this, but no specific plans yet, *N *no, *M *Missing

### Implementation outcomes

#### Reach

Respondents from nearly all trials reported measuring reach (25/29, 86%). Survey respondents operationalized reach as patient enrollment (i.e., percentage of those eligible who enrolled) and/or receipt of a “minimum dose” of the intervention. Less commonly used measures of reach included the percentage of eligible patients offered a referral to the intervention and the percentage who did not opt out of receiving it. Among the planning phase trials, one trial representative indicated an interest in measuring reach but had no specific plans to do so yet.

The most common variables collected to assess representativeness of reach were race/ethnicity (48%, 14/29), sex or gender (31%, 9/29), age (28%, 8/29), and insurance status (21%, 6/29).

#### Adherence

Representatives from most trials (23/29, 79%) reported measuring adherence to or engagement in the study intervention. Of those, some used a patient-level measure of “implementation” based on RE-AIM [19, 24] (i.e., some measured the patient’s adherence to the intervention)—including 71% (5/7) of completed trials, 36% (3/13) of ongoing trials, and 78% (7/9) of pilot phase trials.

#### Adoption

More than a third (41%, 12/29) of respondents reported measuring adoption as part of their trials, including 71% (5/7) of completed trials, 36% (3/13) of ongoing trials, and 33% (3/9) of planning phase trials. About a third of respondents (10/29) measured adoption at the organization level, 10% (3/29) at the clinician level, and 1 at both the organization and clinician levels. Very few (2/29) reported assessing the representativeness of participating clinicians or organizations as part of adoption.

#### Intervention fidelity and adaptations

Nearly all trials, (27/29, 93%) measured intervention fidelity [25, 26], with common assessment methods including direct observation (9/29), checklists (9/29), self-report (11/29), and through medical record data (12/29). Trials were allowed to “check” all that apply, and many of the ongoing and early phase trials measured fidelity across multiple intervention elements. Intervention adaptations and tailoring were measured by 69% of trials (20/29), with data collected via a logbook (10/29; 34%), self-report (7/29; 24%), and/or direct observation (5/29; 17%). Thirteen trials (45%) used an established framework to guide its fidelity/adaptation assessment process, including 4 that incorporated the Framework for Reporting Adaptations and Modifications to Evidence-Based Interventions (FRAME) [27], 2 that applied the Core Function/Form approach [28] and 1 that applied the Core Components and Adaptable Periphery approach [29].

#### Sustainability and sustainment

Only 7 out of 29 trials reported assessing sustainability—defined as the potential for an intervention to be maintained over time—including 2 completed, 2 ongoing, and 3 planning-phase studies. Among the 6 remaining planning-phase trials, all respondents expressed interest in assessing sustainability but had not yet defined a specific approach. Of the 7 trials that assessed sustainability, 3 reported using implementation frameworks to guide this assessment: PRISM (n = 2) and RE-AIM (n = 1). Four trials described using qualitative interviews to explore perceptions of sustainability.

In contrast, sustainment—defined as the continued use of the intervention following the conclusion of the initial implementation or trial—was assessed by 10 of the 29 trials (4 completed, 4 ongoing, and 2 planning phase). Most of the remaining planning-phase studies reported intentions to assess sustainment but had not finalized measurement strategies.

Of the 10 trials that assessed sustainment, five described direct observation of continued intervention delivery, and six reported asking health system decision-makers about ongoing use. One respondent indicated using a national guideline recommendation for continued use of the intervention (or similar models) as evidence of sustainment. Only one trial explicitly noted the timeframe for assessing sustainment (3–6 months after trial completion).

#### Costs

Only 10 of the 29 trials reported measuring any costs associated with intervention delivery, with 2 conducting cost-effectiveness analyses.

#### Barriers and facilitators

Nearly all trials (93%, 27/29) collected data on barriers and facilitators of implementation. Respondents reported using quantitative (41%, 11/27) and qualitative methods (89%, 24/27) to measure implementation determinants.

The survey also asked respondents whether they used specific implementation frameworks to guide the measurement of implementation determinants. Approximately half (13 of 27) reported using the RE-AIM framework for this purpose. Perspectives of clinical and operational leaders regarding barriers and facilitators to intervention delivery were measured in 19 of 27 studies (70%). Twenty studies (74%) gathered data from clinicians actively delivering the intervention, while 9 (33%) captured views of clinicians not directly involved in intervention delivery. Eighteen studies (67%) collected patient perspectives.

In addition, 8 studies (30%) measured the perspectives of other partner groups who, while not always directly delivering care, were considered relevant to implementation success. These included community members and advocates, informatics staff (e.g., responsible for adapting EHR workflows), executive-level health system leaders (e.g., decision-makers for system-wide scale-up), and non-clinical staff delivering the intervention (e.g., community health workers, navigators, or front-desk personnel). Respondents reported that these individuals were included because they may play critical roles in sustaining or expanding the intervention beyond the trial phase, especially if outcomes are favorable. Understanding their views helps anticipate potential barriers or facilitators to broader implementation and sustainment.

## Discussion

Systematic collection of data about implementation processes and outcomes within an ePCT can help strengthen interventions and their effectiveness as well as inform efforts to implement the interventions in future ePCTs and to facilitate their successful sustainment, scale-up, and spread in routine care settings. We observed significant variation in implementation-focused data collection efforts in Collaboratory ePCTs, with many exemplary approaches and best practices applied. For example, survey respondents for nearly all ePCTs reported measuring the reach of the intervention being tested in the trial as the percentage of patients eligible to receive it, instead of simply reporting the absolute number who participated.

Nearly all trials reported assessing intervention fidelity, which is critical for interpretation of primary effectiveness results in ePCTs, as well as efforts to facilitate implementation, sustainment, and scale-up/spread. Attention to intervention adaptation and tailoring was less consistent. According to emerging best practices and the Patient-Centered Outcomes Research Institute (PCORI) Standards for Complex Health Interventions, investigators should consider both Forms and Functions of interventions, along with potential modifications during the design or planning phase of a trial, and anticipated future adaptations [30]. Tracking and reporting intervention adaptations along with contextual reasons for adaptations is important to inform sustainability and future scale, both at the local health system and to other health care systems. While an ePCT may not be designed to explicitly incorporate adaptations, unplanned adaptations can arise, and both planned and unplanned adaptations should be reported. Adaptations of implementation strategies are equally important. Given the complex settings within which ePCTs are conducted and how rapidly the settings change, adaptations should be expected to ensure continued alignment with the context but must be monitored to ensure both rigor of the intervention and to foster sustainability and scalability. Using approaches such as Form and Function assists ePCTs in balancing the need for both adaptations and fidelity [28].

Moreover, representatives from nearly all trials reported assessing implementation determinants (contextual factors; barriers and facilitators), primarily using qualitative methods (e.g., semi-structured interviews). These assessments can be informative for subsequent development and testing of responsive implementation strategies designed to improve suboptimal implementation outcomes such as reach, adoption, or anticipated sustainability at the current setting or when scaling to other settings. An understanding of determinants is also useful for design of future ePCTs that seek to implement and test similar interventions in ways that are adapted for the different contextual issues of a different setting. With most Collaboratory ePCTs already assessing implementation determinants, there may be an opportunity to identify some best practices with respect to timing of data collection and/or data collection instruments for assessing determinants. Further, assessments could be more targeted to specific implementation objectives depending on the stage, setting, and context of the trial. Future work could begin across all projects or in subsets of projects with a similar focus (e.g., NIH Helping End Addiction Long-Term® (HEAL) Initiative trials focused on nonpharmacologic pain management interventions) [31], but at a minimum should encourage iterative assessment given that the context dynamically changes overtime.

In terms of opportunities for growth, only a handful of Collaboratory ePCTs reported assessment of clinician-level adoption, including information about the percentage and representativeness of clinicians who used study interventions. The lack of assessment of clinician-level adoption may reflect heterogeneity of interventions tested in Collaboratory ePCTs or changes to the original definition from the RE-AIM framework. Some interventions require more active “adoption” on the part of a clinician (e.g., making a referral), while many do not. Clinician-level adoption may not be relevant for an ePCT testing an interruptive clinical decision support tool or alert. In this scenario, clinicians do not have a choice to use the intervention but do have a choice of how they respond to it when it interrupts them with a recommendation. This also represents an example where the original definition of clinician adoption from the RE-AIM framework may be modified to reflect not if but how a clinician responds to an intervention [32].

By contrast, computerized provider order entry (CPOE) and electronic health record (EHR) interventions offer unique, natural opportunities for efficient, programmed capture of implementation and compliance data a priori, during the study design phase. In this circumstance, the study intervention design itself can be leveraged to standardize data collection and auto-program it throughout the intervention period, offering unique opportunities for real-time evaluation of compliance and clinician data. This in turn allows investigators to identify implementation and adherence issues proactively and refine them. However, capturing those data in a complete and accurate way can be challenging. In the example of a CPOE intervention like INSPIRE, identifying the ordering physician was complicated by circumstances where orders were entered by persons other than the attending physician (e.g., trainees or nurses). Moreover, large health systems and community hospitals often work with contracted physicians whose personal identifying information can be considered confidential and not extractable by third parties for research purposes due to medical, legal, or other concerns.

A key aspect of promoting equity involves measuring representativeness among patients and providers, although data on gender, race, ethnicity, age, years in practice, etc., are often not available for clinicians within existing data sources [33]. Thus, measuring representativeness of clinician-level adoption is challenging when relying on EHR data given clinician-level characteristics are often stored separately from routinely collected patient or visit-level data and are not always accessible to evaluations. Often provider level data requires primary data collection, which is difficult within the context of ePCTs that aim to rely on routinely collected data. Additionally, better access to high-quality and comprehensive data from the EHR is paramount for ePCTs and the development of Learning Health Systems, not only to measure representativeness at the patient and provider levels, but also to ensure trustworthy results across all outcomes [34, 35].

Most trials did not assess anticipated sustainability or sustainment, representing another opportunity for growth and future funding. Such information is vital for understanding the viability of sustained adoption of an intervention in a non-trial setting, offering critical information about factors that might limit that potential. Collection of such information during a trial gives investigators the opportunity to consider adaptations to the intervention and implementation strategies that might improve the potential to be sustained post-trial. One tool that can be used for such measurements is the Program Sustainability Assessment Tool (PSAT), which was developed to help teams understand an intervention’s (or a public health program’s) capacity for sustainability [36, 37]. Although there is no empirical evidence that PSAT directly predicts sustainability, it can be used as both a measure of sustainability and a planning tool to help optimize sustainability. There is an opportunity for our future research projects to explore anticipated sustainability through the use of tools such as the PSAT [38]. The goal of ePCTs is to improve the translation of evidence into practice, not just for the life of the grant period, but beyond; thus, assessing and planning for sustainability is paramount.

Actual sustainment of an intervention after the conclusion of the trial was more commonly measured across Collaboratory ePCTs. Measuring sustainment is more straightforward for some trials versus others; for example, tracking sustained use with data from the EHR is easier than having to collect primary data from clinicians or other partners.

A major barrier to measuring actual sustainment is a lack of funding for collecting data on sustainment over a longer period of time. NIH does have separate funding mechanisms focused on sustainment and scale up [39], but there may be a significant delay between the end of an ePCT and funding of a subsequent project focused on sustainment of the same intervention.

This study has several limitations and identifies multiple areas for future research. Because the survey was intentionally concise to reduce respondent burden, we focused on a limited set of implementation outcomes (e.g., we did not explicitly ask about acceptability). In addition, deeper assessment of measurement methods was not feasible. For example, we did not ask what tools were used to measure outcomes. The scope of this paper was focused on whether key implementation outcomes were being considered, rather than on how these outcomes were measured, which limits our ability to assess or compare the rigor of measurement approaches across studies. The survey did include open-ended responses about frameworks to solicit other frameworks.

The survey also did not collect detailed information about assessing implementation determinants. A better understanding of which determinants were assessed (e.g., determinants of initial adoption vs. sustainment), and how (e.g., sequential mixed methods) is an important area for future exploration. Future research with a larger sample of studies could also examine measurement of combinations of multiple implementation outcomes and explore relationships between them. These findings also may not directly generalize to other non-Collaboratory ePCTs.

Results from our survey raise an important question: Considering the significant investment required to conduct an ePCT, should potential funding agencies consider requirements for ePCTs to include plans to measure a minimum set of implementation constructs, including the potential to be sustainable? The goal of generating this information could facilitate intervention implementation in future ePCTs and result in the development of implementation strategies that increase the likelihood that effective interventions will translate into practice in ways that are equitable, sustainable, and scalable. If a minimum set of constructs were required, which ones would provide the most actionable information? As ePCT teams are facing an increasing number of requirements, such as sharing data, phenotypes, and meta data; harmonizing data to common data models; and reporting to patients, health care systems leaders, and the scientific community, the question of which constructs to consider becomes especially important so as not to overburden teams. Some efforts already exist, including efforts to create standard data elements within specific domains [40], which may form the foundation for work including aspects that need to be domain specific and aspects that can be more generalized across domains. Ideally, some requirements could be de-implemented to avoid overcomplicating pragmatic trials and inadvertently making them less pragmatic. Evidence reviews and consensus-building activities could help to elucidate the most “actionable” implementation data that could be required, along with best practices in measurement and the extent to which that data needs to be harmonized across ePCTs (e.g., common data elements). Such recommendations should allow for flexibility to both minimize the increasing requirement burdens imposed on pragmatic trials and to ensure they are able to be tailored to the trial’s objectives and context in ways that are meaningful. Given the heterogeneity of pragmatic trial objectives and contexts, harmonization efforts must not be rigid. Ideally, some requirements could be de-implemented to avoid overcomplicating pragmatic trials and inadvertently making them less pragmatic. Moreover, researchers may benefit from existing repositories [41] of assessment instruments in implementation science to facilitate both selection and data harmonization across multiple trials.

## Conclusion

Across 29 ePCTs surveyed, implementation is being assessed in a variety of ways, but measures to improve the consistency and comprehensiveness of evaluating implementation are needed to maximize the real-world impact of evidence-based interventions. Specific aspects of implementation to prioritize are those that improve measurement of the representativeness of clinicians, sustainability and scalability of ePCT interventions.

## Supplementary Information

Supplementary Material 1.

## Data Availability

Data are available upon reasonable request from the corresponding author at devonche@med.umich.edu.

## Abbreviations

CSAT: Clinical Sustainability Assessment Tool
CPOE: Computerized provider order entry
CFIR: Consolidated Framework for Implementation Research
EHR: Electronic health record
ePCTs: Embedded pragmatic clinical trials
EPIS: Exploration, Preparation, Implementation, Sustainment
FRAME: Framework for Reporting Adaptations and Modifications to Evidence-Based Interventions
FOAs: Funding Opportunity Announcements
HEAL: Helping to End Addiction Long-Term^®^
IRB: Institutional Review Board
NIH: National Institutes of Health
N/A: Not applicable
NOFOs: Notice of Funding Opportunities
PCORI: Patient-Centered Outcomes Research Institute
PRISM: Practical, Robust Implementation and Sustainability Model
PIs: Principal Investigators
PSAT: Program Sustainability Assessment Tool
PARiHS: Promoting Action in Research Implementation in Health Services
RE-AIM: Reach, Effectiveness, Adoption, Implementation and Maintenance
SMSS: Sustainment Measurement System Scale
TSBM: Translational Science Benefits Model
