# Supplementary material for: Collection of implementation-related data in pragmatic clinical trials: a cross-sectional study from the NIH Pragmatic Trials Collaboratory
Source: Implement Sci Commun. 2026 Feb 13;7:48. doi: 10.1186/s43058-026-00868-4 (PMC13005506; doi:10.1186/s43058-026-00868-4)
Supplement: Supplementary file 1 — Supplementary Material 1. [file 43058_2026_868_MOESM1_ESM.docx]

**Appendix 1 Survey**


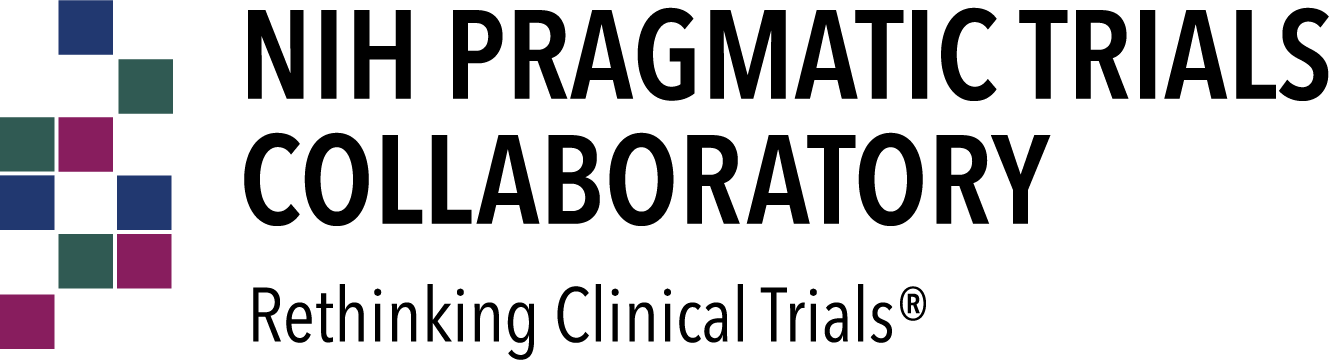


Default Question Block

1. What is your name?
2. What is the name of the Collaboratory Demonstration Project for which you are completing this survey?
3. In your Collaboratory Demonstration Project, are you measuring or planning to measure **fidelity** (clinician’s delivery of an intervention as intended)?


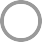

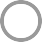
 Yes No

1. If you are measuring **fidelity**, how are you measuring it? [select all that apply]


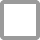
 Direct observation
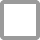
 Checklists


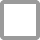
 Self-report


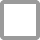
 Medical record data


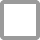
Other (fill-in below)


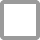
N/A; we are not measuring fidelity

1. In your Collaboratory Demonstration Project, are you measuring or planning to measure **adaptations** (changes or modifications to an intervention)?


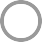

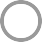
 Yes No

1. If you are, how are you measuring **adaptations**? [select all that apply]


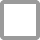
 Direct observation
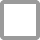
 Checklists


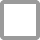
 Self-report


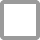
Recording adaptations in a log


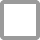

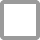
Other (fill-in below) N/A; we are not measuring adaptations

1. Are any of the following frameworks or concepts informing your assessment of **fidelity and/or adaptations**? [select all that apply]


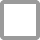
 Core Forms and Functions


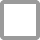
 Core Components and Adaptable Periphery


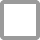
 Framework for Adaptation and Modification (FRAME)


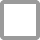
Other (fill-in below)


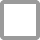
 N/A; we are not measuring fidelity and/or adaptations


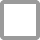
 N/A; we are measuring fidelity and/or adaptations, but we are not using a framework

1. In your Collaboratory Demonstration Project, are you measuring or planning to measure **anticipated sustainability** (potential for an intervention to be maintained or institutionalized after a trial concludes)


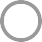

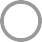
 Yes No

1. If you are measuring **anticipated sustainability**, how are you measuring it? [select all that apply]


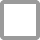
 Program Sustainability Assessment Tool (PSAT) or Clinical Sustainability Assessment Tool (CSAT)


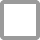
 Sustainment Measurement System Scale (SMSS)


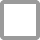
Other (fill in below)


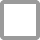
N/A; we are not measuring anticipated sustainability

1. In your Collaboratory Demonstration Project, are you planning to measure **known/actual sustainment** (actual maintenance or institutionalization of an intervention after a trial concludes)?


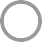

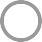
 Yes No

1. If you are measuring **known/actual sustainment**, please provide more detail about your plans (e.g., any specific measures of sustainment, timeframe for assessing sustainment, who in the target health systems will provide information about sustainment).
2. Are you assessing (or planning to assess) **barriers to and/or facilitators of implementing** (i.e., delivering, using, engaging with) **the intervention** in the context of the trial?


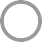

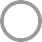
 Yes No

1. If you are assessing **barriers to and/or facilitators of implementing the intervention**, what methods are you using? [select all that apply]


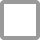
 Quantitative
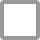
 Qualitative


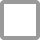
Other (fill-in below)


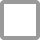
N/A; we are not measuring barriers/facilitators

1. If you are assessing **barriers to and/or facilitators of implementing the intervention**, what framework(s) are guiding data collection related to barriers and facilitators? [select all that apply]


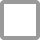
 Consolidated Framework for Implementation Research (CFIR)


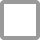
 Promoting Action on Research Implementation in Health Services (PARIHS)


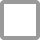
 Exploration, Preparation, Implementation, Sustainment (EPIS)


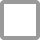
 Practical, Robust Implementation and Sustainability Model (PRISM, which include RE-AIM)


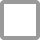
 Reach, Effectiveness, Adoption, Implementation, Maintenance (RE-AIM)
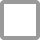
 Other (fill in below)


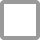
 N/A; we are not measuring barriers/facilitators


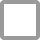
N/A; we are measuring barriers/facilitators, but we are not using a framework

1. If you are assessing **barriers to and/or facilitators of implementing the intervention**, from what perspective(s) are you assessing barriers and facilitators? [select all that apply]


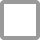
 Clinical operations leaders


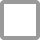
 Clinicians delivering the intervention


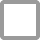

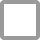
 Clinicians not delivering the intervention Patients


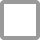

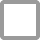
Other (fill-in below) N/A; we are not measuring barriers/facilitators

1. In your Collaboratory Demonstration Project, are you measuring or planning to measure **reach** (number and percent of eligible patients who participate in an intervention and the representativeness of those patients)?


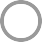

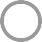
 Yes No

1. If you are measuring **reach**, how are you defining participation [select all that apply]?


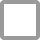
 Study enrollment


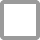
 Receipt of a “minimum dose” of the intervention


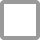

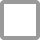
Other (fill-in below) N/A; we are not measuring reach

1. If you are measuring reach, are you assessing the representativeness of patients who participate in the intervention? If so, what characteristics are you considering (e.g., race/ethnicity, insurance status, age)? If you are not measuring reach, please write N/A.
2. In your Collaboratory Demonstration Project, are you measuring or planning to measure **adoption** (number and percent of eligible organizations or clinicians that decide to take up or use an intervention)?


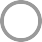

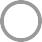
 Yes No

1. If you are measuring **adoption**, at what level are you measuring it [select all that apply]?

- Organization level
- Clinician level
- Other (fill-in below)
- N/A; we are not measuring adoption

1. If you are measuring **adoption**, how are you defining uptake or use of the intervention (e.g., decision to participate in the trial, providing the intervention to some or all eligible patients)? If you are not measuring adoption, please write N/A.
2. If you are measuring **adoption**, are you assessing the representativeness of organizations or clinicians that adopt the intervention? If so, what characteristics are you considering? If you are not measuring adoption, please write N/A.
3. In your Collaboratory Demonstration Project, are you measuring or planning to measure **patients’ engagement in or adherence to the intervention**?


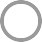

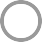
 Yes No

1. If you are measuring **patients’ engagement in or adherence to the intervention**, are you assessing differences in engagement or adherence based on patient characteristics? If so, what characteristics? If you are not measuring this, please write N/A.
2. Does your trial use a hybrid effectiveness/implementation design? If yes, what type?


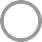
 Type I: Focuses primarily on effectiveness outcomes of an intervention while exploring the “implementability” of an intervention, usually through an implementation-focused process evaluation


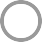
 Type II: Simultaneous testing or piloting of implementation strategies during an effectiveness trial


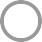
 Type III: Focuses primarily on implementation outcomes while also collecting effectiveness outcomes as they relate to adoption or fidelity of an intervention


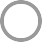
N/A; our trial does not use a hybrid design

1. Are you collecting data on costs associated with the intervention? If yes, please describe.
2. Are you using the [Translational Science Benefits Model](https://translationalsciencebenefits.wustl.edu/) [(TSBM)](https://translationalsciencebenefits.wustl.edu/) to assess the impact of your project?
3. What additional information you would like to share regarding implementation-related aspects of your project?
